# A Case of Plummer-Vinson Syndrome in an Omani Woman

**DOI:** 10.7759/cureus.41050

**Published:** 2023-06-27

**Authors:** Alhasan Asaad, Rama Jamal

**Affiliations:** 1 Department of Internal Medicine, National University of Science and Technology, Muscat, OMN

**Keywords:** anemia, dysphagia, esophageal web, iron deficiency anemia (ida), plummer-vinson syndrome

## Abstract

Plummer-Vinson syndrome (PVS) is the triad of iron-deficiency anemia, esophageal webs and dysphagia. This article discusses the first reported case of PVS from Oman. A female patient in her 40s presented with a one-year history of dysphagia and odynophagia and a known background of untreated iron-deficiency anemia. After an elaborate workup, a diagnosis of PVS was made following visualization of the esophageal web with barium swallow study and esophagogastroduodenoscopy (EGD). A prompt and accurate diagnosis of PVS, although rare, is crucial, given that it is, at times, a precancerous condition. The treatment with iron therapy is the cornerstone of management, and it corrects the anemia as well as the dysphagia. If dysphagia persists, endoscopic dilation can be carried out.

## Introduction

Plummer-Vinson syndrome (PVS) is a rare disorder characterized by the classic triad of iron-deficiency anemia, dysphagia and esophageal webs. It is named after two American physicians, Dr. Henry Stanley Plummer and Dr. Porter Paisley Vinson, who had first described the syndrome. In the United Kingdom, PVS may be termed as Kelly-Paterson syndrome, after two British physicians, Dr. Adam Brown-Kelly and Dr. Donald Ross Paterson, who published their findings later in 1919 [1-4].

PVS mainly affects women from Europe and North America in their fourth to seventh decade. Patients usually present with dysphagia or symptoms and signs of iron-deficiency anemia. On gross pathology, esophageal webs are characteristic findings of PVS. Histopathological analysis reveals epithelial atrophy or hypertrophy, fibrosis, chronic submucosal inflammation and epithelial atypia or dysplasia in advanced cases [1,5].

Esophageal webs are thin mucosal membranes that grow across the esophageal lumen and may become obstructive, causing dysphagia. The presence of esophageal webs alone is not diagnostic of PVS as they can be asymptomatic or not accompanied by iron-deficiency anemia [1,6].

The exact pathophysiology of PVS and the formation of esophageal webs remain largely unknown. However, the most widely accepted hypothesis suggests that chronic iron-deficiency anemia plays a major role. It is postulated that iron deficiency can induce oxidative stress and subsequent DNA damage in the epithelia of esophageal mucosa predisposing to the formation of esophageal webs. Moreover, those webs tend to occur in the post-cricoid region, the area that experiences maximum trauma during swallowing, explaining the increased risk of web formation [1,7,8].

## Case presentation

An Omani female in the fourth decade of her life presented to the outpatient Internal Medicine Department at Sohar Hospital with complaints of difficulty and painful swallowing (odynophagia) for one year. The dysphagia was of gradual onset, and progressive, starting as difficulty swallowing solids, which then recently developed into difficulty swallowing both solids and liquids with feelings of food and drinks “being stuck in her throat”. She reported having easy fatiguability as well as unintentional weight loss but denied any fever, abdominal pain, nausea, vomiting, heartburn, hematemesis, melena, joint pain, skin rashes or Raynaud’s phenomenon. The patient gave a history of chronic iron-deficiency anemia of several years and has not taken iron supplements previously. The patient denied any abnormalities in the menstrual cycle and had no pregnancies in the past. The surgical history is unremarkable. A family history of G6PD deficiency was reported to be present in siblings. No family history of gastrointestinal malignancy or autoimmune disorders is present.

On examination, the patient appeared tired but was fully conscious and cooperative. The patient was vitally stable. Conjunctival pallor, glossitis and angular stomatitis were noted. On hand examination, the patient also had koilonychia and pallor. No lymphadenopathy or skin rashes were seen. On abdominal examination, no tenderness or organomegaly was present, but a round, firm, smooth mass (diameter of 3 cm approximately) over the lower abdomen was palpated. It was immobile and non-tender.

Laboratory studies on admission were suggestive of microcytic hypochromic anemia secondary to iron deficiency. No leukocytosis or leukopenia was noted. Thyroid, liver and renal function tests were all within normal levels. Autoimmune workup (including rheumatoid factor, anti-nuclear antibody, erythrocyte sedimentation rate and antibodies specific to Sjogren's syndrome and scleroderma) was unremarkable as well (Table 1).

**Table 1:** Relevant lab findings on admission Hb: hemoglobin, Hct: hematocrit, MCV: mean corpuscular volume, MCH: mean corpuscular hemoglobin, MCHC: mean corpuscular hemoglobin concentration, RDW: red cell distribution width, MPV: mean platelet volume, TIBC: total iron-binding capacity.

Parameter	Reading	Reference range
WBC	3.57 x 10^3 ^/µL	2.4-9.5 x 10^3 ^/µL
RBC	3.81 x 10^6 ^/µL	4.1-5.4 x 10^6 ^/µL
Hb	6.10 g/dL	11-14.5 g/dL
Hct	19.90	34-43
MCV	52.20 fL	78-95 fL
MCH	16.00 pg	26-33 pg
MCHC	30.70 g/dL	31-35 g/dL
RDW	14.30%	11.5%-16.5%
Platelets	246 x 10^3 ^/µL	150-450 x 10^3 ^/µL
MPV	9.76 fL	7-10.5 fL
Iron	2.67 µmol/L	5.83-34.5 µmol/L
Ferritin	2.38 µg/L	13-150 µg/L
TIBC	78.97 µmol/L	
% Transferrin saturation	3.38%	20%-50%

**Figure 1:**
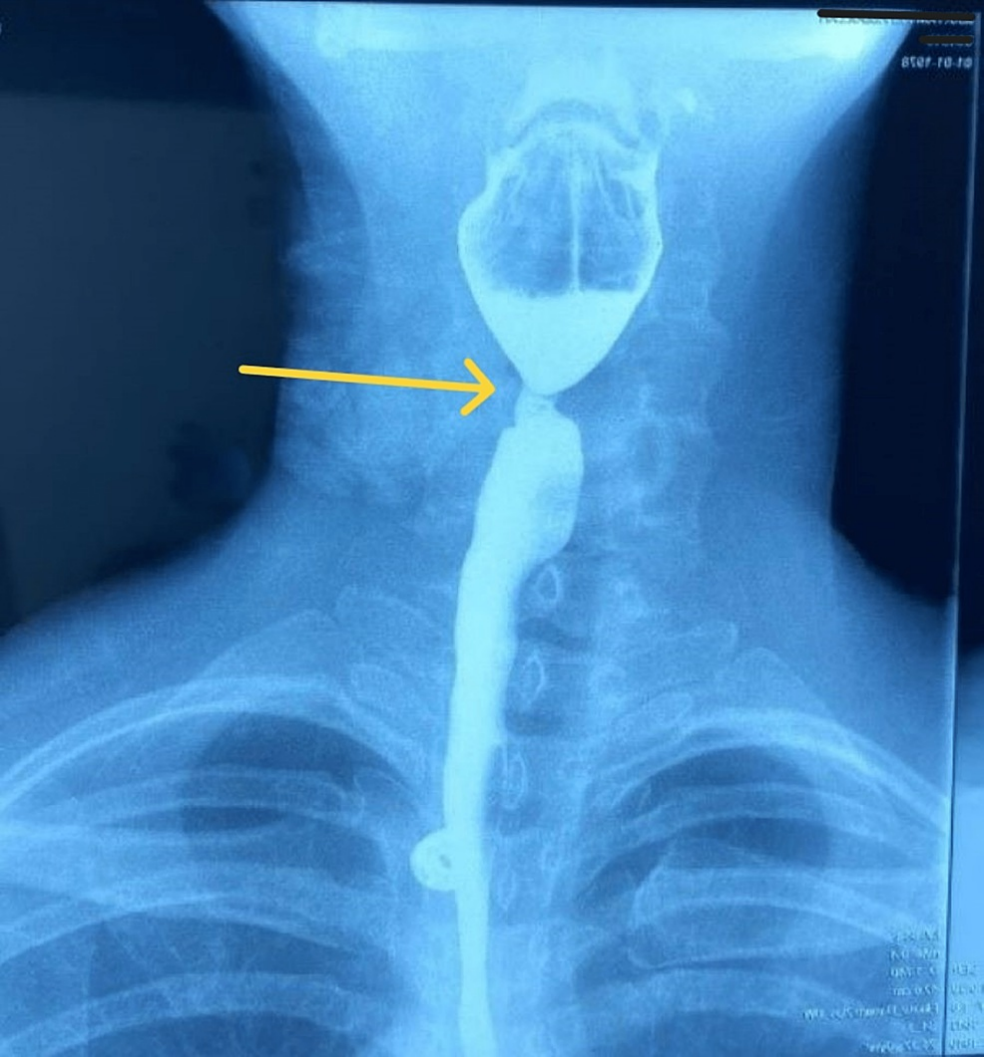
Barium swallow study done during the admission (anterior view), which revealed persistent narrowing (arrow)

**Figure 2:**
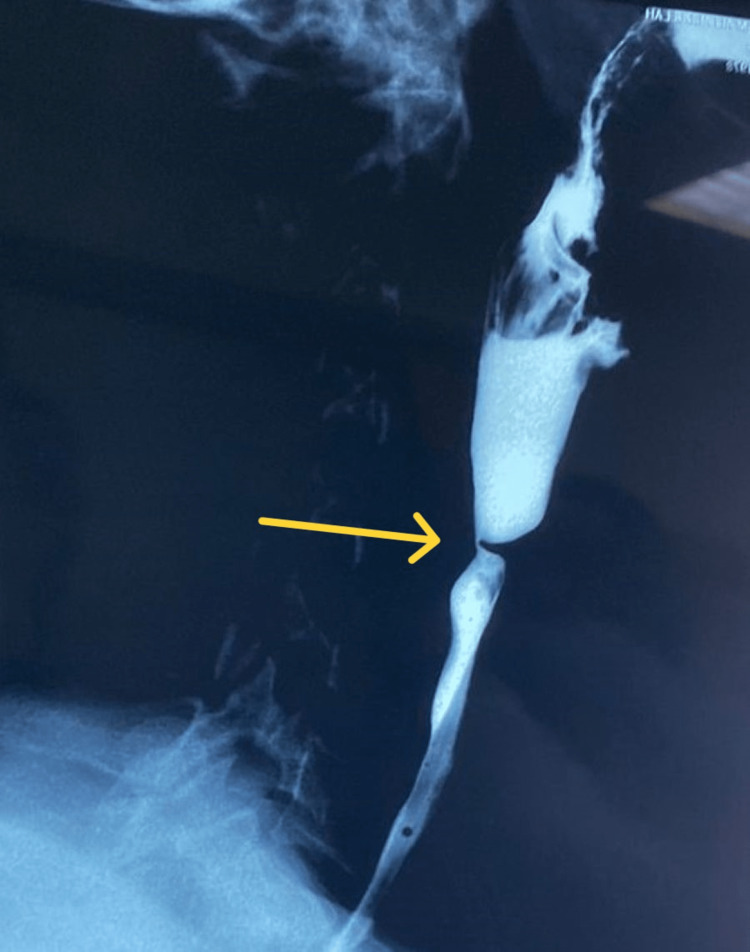
Barium swallow study done during admission (lateral view), showing persistent narrowing seen at the proximal esophagus (arrow) with web formation, with contrast freely flowing into the stomach and small bowel loops

Subsequently, a barium swallow study was performed at the Radiology Department of Sohar Hospital, which revealed persistent narrowing in the proximal esophagus with web in the absence of complete obstruction (Figures 1, 2). Ultrasound of the neck and abdomen was unremarkable. Ultrasound of the pelvis revealed a uterine fibroid measuring 5 × 5.5 cm. The patient then underwent an esophagogastroduodenoscopy (EGD) that confirmed the presence of the esophageal web adjacent to the upper esophageal sphincter which was ruptured via the scope. A biopsy sample of the esophageal web was taken for the histopathology department and later revealed no malignant changes.

The patient received one unit of packed red blood cells, and her hemoglobin was 7.8 g/dL on day 1 post-transfusion. Dysphagia and odynophagia both improved greatly post-EGD. The patient was started on intravenous iron therapy during the same admission and was given follow-up appointments at the Gastroenterology and Gynecology Departments in Sohar Hospital, as well as an appointment at the Day Care Department in Sohar Hospital for intravenous iron therapy after two weeks. Additionally, she was counseled on dietary modifications, and regular iron supplements were prescribed to be started after iron intravenous therapy.

## Discussion

With over a century since the first description of Plummer-Vinson syndrome, or Kelly-Patterson syndrome, our knowledge is limited mostly to its clinical presentation, and most of the literature available consists of case reports and case series. Following our literature review, we found that this case is the first case reported from Oman, along with other few reports from the nearby region; although epidemiologically, we know that the syndrome mostly affects North Americans and Europeans [9-11]. Our patient presented with the classical presentation of the syndrome's triad of iron-deficiency anemia, dysphagia and esophageal webs and was managed successfully with iron supplementation and endoscopic dilation. One theory suggests that esophageal web formation may be caused by iron deficiency leading to mucosal degeneration due to the dysfunction of certain iron-dependent enzymes [12]. However, a review of 1,000 radiographic images of the upper esophagus found that 5.5% had one or more esophageal webs, but interestingly, none of the patients met the diagnostic criteria of PVS and the prevalence of iron-deficiency anemia was similar to that in the control group [13]. Moreover, in another study of 135 patients with confirmed PVS and esophageal webs through EGD, only 38.5% were confirmed to have iron-deficiency anemia, and 0.7% had low serum ferritin without anemia. These studies indicate that iron-deficiency anemia may not fully explain the development of esophageal webs and PVS. In the same study, which is the largest case series available on PVS, the outcomes of patients with PVS were favorable. After a mean follow-up period of 30.7 months, total resolution of dysphagia was observed in 69%, recurrence was observed in 7.3% and malignant transformation only in 3.7% of the cases [14]. Other studies explored the association and risk of malignancy in relation to PVS, making screening for malignancy in these patients after diagnosis and follow-up mandatory [15].

## Conclusions

Due to its rarity, the diagnosis of PVS can sometimes be missed. Additionally, while PVS outcomes have greatly improved after the introduction of therapeutic endoscopy, the pathophysiology remains under investigation. Moreover, the link between PVS and upper gastrointestinal carcinoma remains, somewhat, unclear until now, and screening for malignancy is mandatory. In light of these issues, a systematic approach to data collection and management of these patients is needed in order to improve our understanding of this syndrome and its implications.
